# Effect of Calcitriol and Vitamin D Receptor Modulator 2 on Recovery of Injured Skeletal Muscle in Wistar Rats

**DOI:** 10.3390/biomedicines11092477

**Published:** 2023-09-07

**Authors:** Ioannis Stratos, Svenja Schleese, Ingmar Rinas, Brigitte Vollmar, Thomas Mittlmeier

**Affiliations:** 1 Department of Orthopaedic Surgery, University of Wuerzburg, 97074 Wuerzburg, Germany; 2 Department of Trauma, Hand and Reconstructive Surgery, University of Rostock, 18057 Rostock, Germany; 3 Institute for Experimental Surgery, University of Rostock, 18057 Rostock, Germany; brigitte.vollmar@med.uni-rostock.de

**Keywords:** muscle injury, calcitriol, vitamin D receptor modulator 2 (VDRM2), healing, cellular turnover

## Abstract

Muscle injuries often result in functional limitations due to insufficient healing. This study assessed the influence of calcitriol and vitamin D Receptor Modulator 2 (VDRM2) on muscle regeneration in male Wistar rats following open blunt muscle injury. The injured left soleus muscle of the rats was treated for the first four days after trauma with local injections of either calcitriol, VDRM2, or a 10% ethanol solution (control). Although muscle strength significantly decreased post-injury, all groups showed gradual improvement but did not achieve full recovery. By the 14th day, calcitriol-treated rats significantly outperformed the control group in the incomplete tetanic force, with VDRM2-treated rats showing muscle strength values that fell between the control and calcitriol groups. Similar trends were observed in complete tetanic contractions and were confirmed histologically via muscle cell width quantification. Additionally, histological analysis showed increased cellular turnover on the fourth postoperative day in the calcitriol group, as indicated by elevated cell proliferation rates and fewer apoptotic cells. VDRM2-treated animals showed only an increased proliferative activity on day 4 after injury. No noticeable differences between the groups for CAE-positive cells or visible muscle tissue area were found. In conclusion, predominantly calcitriol positively influenced post-trauma muscle recovery, where VDRM2 had substantially lower biological activity.

## 1. Introduction

In the fields of sports medicine and trauma surgery, muscle injuries—frequently attributable to overuse, direct injury, or fractures—are prevalent, resulting in pain, dysfunction, and compromised performance. The evolution of medical, pharmacological, and physical therapeutic interventions remains crucial for efficient injury prophylaxis and management, markedly enhancing outcomes. Several pharmacological agents are either under investigation or currently employed to facilitate muscle function restoration [1,2]. However, further research is imperative to comprehensively elucidate their efficacy and potential adverse effects.

Vitamin D (calcitriol) is among these pleiotropic substances that can positively influence the muscle tissue upon injury. Previous research in C2C12 mouse myoblasts has shown that the expression of vitamin D receptors (VDR) and the cytochrome P450 27B1 (CYP27B1), an enzyme involved in calcitriol metabolism, increases during muscle regeneration [3]. Maintaining serum levels of calcitriol may be beneficial for enhancing reparative processes and potentially facilitating subsequent hypertrophy in humans [4]. Moreover, calcitriol has been shown to increase cellular turnover upon injury by inhibiting apoptosis and accelerating cellular proliferation in the skeletal muscle of rat [5]. As a result, further research is needed to characterize optimal calcitriol levels, study the efficacy of calcitriol administration, and compare multiple analogs of calcitriol to elucidate its potential as a significant contributor to muscle regeneration following injury [6].

Many vitamin D receptor modulators (VDRMs), distinguished by their absence of calcitriol’s 9,10-steroid structure, have been created to date. These artificial alternatives, including bi- and tri-aromatic triols, podocarboxylic acid derivatives, lumisterol derivatives, and diarylmethanes, are recognized as ligands by the vitamin D receptor (VDR) similar to calcitriol, the original ligand [7,8]. These VDRMs activate analogous transcriptional and signaling pathways as calcitriol, but their effectiveness varies based on their individual chemical characteristics and in comparison to calcitriol [9]. Factors such as hydrophobic or hydrophilic qualities impact their ability to bind to vitamin D-binding proteins (DBPs). The strength of transcription can also be influenced by structural changes to the VDRM backbone [8,9]. Non-secosteroidal ligands are known to be VDR agonists [10]. VDR antagonists and partial agonists are typically based on the secosteroid scaffold of calcitriol, and only a few non-secosteroidal VDR antagonists are known [11].

Non-secosteroidal VDR ligands have been developed for their agonist with non-calcemic profiles [12]. Induction of distinct VDR conformations and cofactor recruitment may be associated with selective actions of non-secosteroidal VDR ligands [13]. The non-secosteroidal vitamin D receptor modulator 2 (VDRM2; LSN2148936), a diarylmethane, is part of this group [9]. An added benefit of VDRM2’s distinctive chemical structure is that it’s not recognized as a substrate by the CYP24A1 enzyme, which typically degrades calcitriol, hence decreasing its effectiveness [14]. VDRM2, as an effective VDR agonist, has its potency varying across different target tissues [14,15]. Studies, including specific RNA analysis, showed an 80% overlap between genes influenced by calcitriol and VDRM2 [14]. Importantly, no hypercalcemic side effects, often associated with calcitriol, have been found with VDRM2 [14,15]. VDRM2 has demonstrated a high safety margin, inducing hypercalcemia in rats [15]. VDRM2 is a non-secosteroidal vitamin D receptor agonist that has been shown to inhibit prostate tumor growth using a xenograft mouse model [14]. Further animal studies have shown that VDRM2 is well tolerated and effective in restoring bone mass, spatial architecture, and bone strength [16].

This study aimed to explore the impact of VDRM2 and calcitriol on the regeneration of peripheral skeletal muscle following trauma. Specifically, we investigated the biomechanical effects on contractile capacity and muscle strength post-trauma following VDRM2 and calcitriol administration and the histological alterations, including apoptosis, proliferation, local myeloid cell infiltration, muscle tissue fraction, and myofiber widths after application of VDRM2 and calcitriol.

## 2. Materials and Methods

### 2.1. Experimental Setup and Groups

All experiments were carried out on male Wistar rats (Charles River Laboratories, Sulzfeld, Germany) weighing 355 ± 36 g. In accordance with the guidelines of the German Animal Welfare Law, the animals were housed in a climate-controlled room at the Institute for Experimental Surgery, University of Rostock. They were kept in individual cages with a 12 h day-night rhythm provided with laboratory rodent feed and water ad libitum. The animals had a one-week acclimation period to these laboratory conditions before experiments were conducted. Approval for the scientific project was obtained from the local authorities (State Agency for Agriculture, Food Safety and Fisheries Mecklenburg-Western Pomerania; Approval Number 7221.3-1.1-048/10-1).

A total of 68 animals underwent left M. soleus injury on day 0. Immediately after the injury, the animals were divided into experimental groups and received four consecutive intraperitoneal injections (i.p.) every 24 h. The injections consisted of either calcitriol, VDRM2, or a 10% ethanol solution, depending on the group assignment. The injections were administered i.p. on days 0, 1, 2, and 3 following the muscle injury. Subsequent analysis of the animals was conducted on days 1, 4, and 14 after the muscle injury.

The study included the following groups:

- The calcitriol used was the activated 7-dehydrocholesterol (catalog number 47763 SUPELCO; Sigma-Aldrich Chemie GmbH, Hamburg, Germany). The VDRM2 was provided upon request from Lilly Research Laboratories (Lilly Research Laboratories, Indianapolis, IN).
- Control group (*n* = 21): The animals in this group underwent muscle trauma induction on day 0. From day 0 to 3, they received a daily i.p. injection of 10% ethanol (2 mL/kg body weight/day).
- Calcitriol group (*n* = 21): The animals in this group underwent muscle trauma induction on day 0. From day 0 to 3, they received a daily i.p. injection of calcitriol (1 μg/kg body weight/day).
- VDRM2 group (*n* = 21): The animals in this group underwent muscle trauma induction on day 0. From day 0 to 3, they received a daily i.p. injection of VDRM2 (1 μg/kg body weight/day).

**Figure 1:**
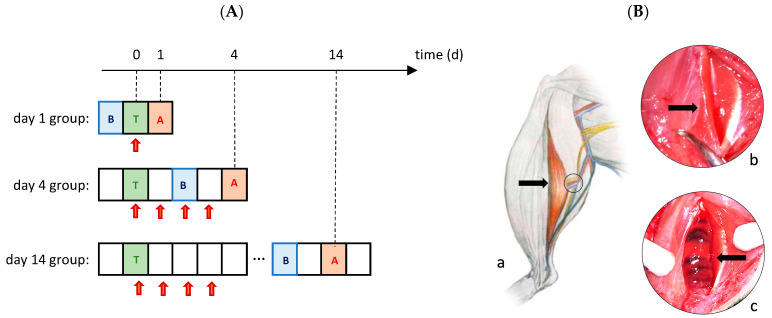
(**A**): Experimental design. Calcitriol, VDRM2, or 10% ethanol solution was injected i.p. on the day of trauma induction (T; green square) and on each of the following three days (red arrows). The final analysis (**A**; brown square) took place on the 1st, 4th, and 14th postoperative days. 48 h before the final analysis, the i.p. injection of 50 mg/kg bw BrdU (**B**; blue square) was applied. (**B**): Graphical representation of the soleus muscle (arrow) with the supplying vessels and nerve (circle) (**a**). Trauma induction of the left soleus muscle is performed using the instrumented clamp (**b**). Shown in (**b**) is the first of the seven contusions. The macroscopic result directly after traumatization of the muscle by seven successive contusions (**c**). The muscle area with the central nerve vessel bundle (arrow) is left out of the trauma (**c**).

In vivo measurements of muscle force, as well as histological and immunohistochemical analysis, were performed on days 1, 4, and 14 after the muscle injury. There were seven animals per time point and per group included in these analyses (Figure 1A).

### 2.2. Muscle Injury

Animals were randomly assigned to different groups and anesthetized with an intraperitoneal injection of 6% Pentobarbital Sodium (55 mg/kg body weight, Narcoren, Merial GmbH, Hallbergmoos, Germany). The left hindlimb was prepared, and the animal was positioned prone on a heating plate (Klaus Effenberger, Medical Devices, Pfaffing, Germany) to maintain a core body temperature of 36–37 °C. Aseptic techniques and microsurgical instruments were used for all procedures. A 2.5 cm incision was made on the hindlimb, and the flexor muscles (M. gastrocnemius/M. soleus) were exposed. The M. soleus was then contused using an instrumented clamp, with the force measured in real-time using the DMC plus instrument (Hottinger Baldwin Messtechnik GmbH, Darmstadt, Germany). The contusion was applied seven times for 10 s each at 25 N over the M. soleus’s length without damaging the central vascular nerve bundle (Figure 1B). The wound was rinsed with saline, sutured with a 4-0 Vicryl suture (Ethicon GmbH, Norderstedt, Germany), and disinfected with Povidone-Iodine. Postoperatively, rats received Novaminsulfon in drinking water for three days and had access to standard feed and water.

### 2.3. Muscle Force Measurement

Muscle force was measured in vivo on the first, fourth, or 14th day post-muscle injury, depending on the experimental group. After anesthesia with 6% Pentobarbital Sodium i.p., both soleus muscles were exposed. The Achilles tendon was detached from the calcaneus and sutured to a digital force gauge. Direct stimulation of the soleus muscle at 9 mA/75 Hz elicited an incomplete tetanic force (stimulation duration: 0.1 s) or a complete tetanic contraction (stimulation duration: 3 s) of the soleus muscle. The first five curves for both the incomplete tetanic and complete tetanic contraction were used to compute the mean of their maximum values.

### 2.4. Histology and Immunohistochemistry

The soleus muscle was excised, fixed in 4% formalin for three days, and embedded in paraffin for histological and immunohistochemical analyses. Longitudinal 4 μm-thick sections were cut from the muscle’s proximal to distal end and transferred onto poly-L-lysine-coated slides. Tissue sections were then deparaffinized using a descending alcohol series.

### 2.5. Muscle Cell Proliferation and BrdU Immunohistochemistry

For the quantification of the muscle cell proliferation, BrdU (50 mg/kg body weight) was intraperitoneally administered to the experimental animals 48 h prior to tissue collection. The incorporation of BrdU into the skeletal muscle cell DNA was used as an immunohistochemical marker for proliferation activity within these muscles, as described and analyzed previously [5].

For the BrdU immunohistochemistry, we used the full-length muscle applied on a poly-L-lysine-coated slide. Antigen retrieval was conducted in a microwave at a pH of 6.0 using DAKO Target Retrieval Solution (S1699, Dako Deutschland GmbH, Hamburg, Germany). Non-specific binding sites and peroxidase activity were blocked utilizing a specialized blocker solution (X0909, DAKO Protein Block, 3% or DAKO Peroxidase Block). The tissue was incubated with a monoclonal anti-BrdU primary antibody (M0744, Dako Deutschland GmbH, Hamburg, Germany) at 4 °C for 18 h. This was performed in a 1:50 dilution with an antibody dilution medium (S3022, Dako Deutschland GmbH, Hamburg, Germany). The secondary antibody employed was a goat anti-mouse IgG/HRP (P0447, Dako Deutschland GmH, Hamburg, Germany), used at a dilution of 1:100 as per the manufacturer’s guidelines. The tissue was then incubated with DAB dye (K3467, 3.3′-diaminobenzidine tetrahydrochloride, Dako Deutschland GmbH, Hamburg, Germany) for approximately 13 min. This step caused the BrdU-positive cell nuclei to stain a brownish color. Tissue sections were counterstained with Mayer’s Hemalum solution for 5 min and dehydrated in an ascending alcohol series (41-52-13, X-TRA-Solv, Medite, Burgdorf, Germany). Finally, tissue sections were coverslipped using X-TRA KITT (41-5219, Medite, Burgdorf, Germany). The muscle was then evaluated in its entirety.

### 2.6. CAE Histology

Analysis of tissue infiltrating mast cells, myeloid cells, and neutrophils were quantified using CAE-staining. Therefore, deparaffinized muscle tissue samples were treated with Naphtol AS-D chloroacetate (NO-758-1g, Sigma Diagnostics, Deisenhofen, Germany), dehydrated in ascending alcohol series, and covered. The leukocyte-specific esterases lead to a hydrolysis reaction of the naphthol AS-D chloroacetate. The resulting visualisation of the naphthol compound shows the infiltrating leukocytes in the soleus muscle.

### 2.7. TUNEL Histology

Apoptotic skeletal muscle cells were identified histomorphologically using the TUNEL method (S7101 ApopTag Kit, Chemicon International Inc., Temecula, CA, USA), which involves attaching a biolabeled dUTP to apoptotic cell DNA fragments. This biotin label is made visible using color-labeled antibodies. Muscle tissue was cut, deparaffinized with X-TRA (Medite, Germany), and rinsed with PBS. Next, the sample underwent Proteinase K digestion for 15 min, followed by a PBS wash, 3% peroxidase treatment, and another PBS wash. Equilibration buffer was applied for 10 s; then samples were incubated for an hour with TdT enzyme buffer (ApopTag kit) in a 37 °C humid chamber. Stop/Wash buffer was applied for 10 min, then PBS wash and 30 min incubation with anti-digoxigenin peroxidase antibody at room temperature. After four washes, samples were incubated with DAB dye for 5 min and washed thrice with PBS. Lastly, counterstaining was performed with Mayer’s Haemalaun nuclear stain for 5 min, dehydration in ascending alcohol series (X-TRA-Solv, Medite, Germany), and coverslipping (X-TRA KITT, Medite, Germany).

### 2.8. Quantification of CAE, BrdU and TUNEL Staining

To objectively evaluate the morphologically heterogeneous traumatized muscle tissue, the entire length of the muscle was observed and analyzed quantitatively. The BrdU, CAE, and TUNEL stains were evaluated under a light microscope (Zeiss Axioskop 40; Göttingen, Germany) at 400× magnification. The M. soleus was examined from its proximal to distal attachment along its complete length, where, on average, 30 fields of view were studied, and the count of positively stained cells was recorded. The arithmetic mean of cells per field of view was calculated and converted into cells per mm^2^.

### 2.9. HE Staining and Analysis of Myofiber Width and Muscle Tissue Area

Utilizing hematoxylin-eosin (HE) stained slides, we were able to examine the structural integrity of the damaged muscular tissue. The HE-staining protocol was executed as follows: Initially, the paraffin-embedded slides were deparaffinized and stained with Mayer’s hematoxylin solution. Post-hematoxylin application, the slides were thoroughly rinsed. They were then counterstained with an Eosin solution. The final stages of the procedure involved dehydration and clearing of the slides, utilizing conventional laboratory methodologies.

**Figure 2:**
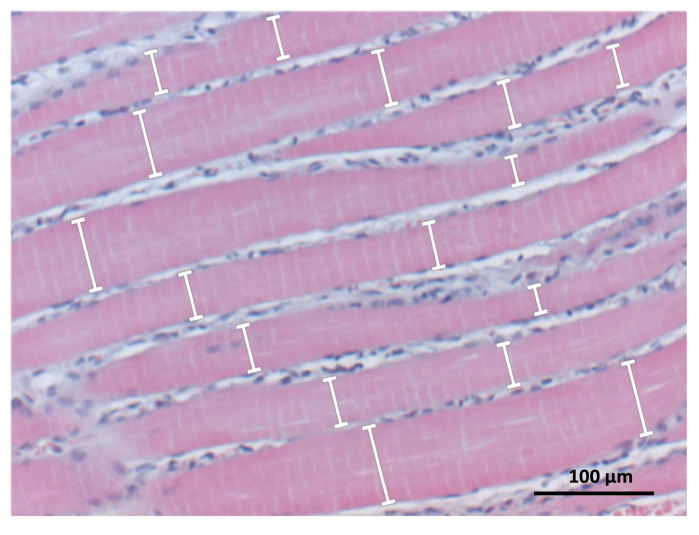
Method for measurement of the muscle fiber width in HE-stained muscle sections. The width of each visible muscle fiber was measured twice at perpendicular points (white arrows with straight ends), recorded in μm, and averaged. Tissue sample: injured soleus muscle; magnification: ×200.

The calculation of the muscle fiber width was assessed In the HE-stained muscle sections. The measurements were performed using Olympus cell^D software (v 2.2; Olympus Soft Imaging Solutions GmbH, Hamburg, Germany). Prior to measurement, the scale was set by defining the relation between pixel and µm using a microscope micrometer. Twelve observation fields per section were measured in the penumbra zone (i.e., the zone between injured and healthy muscle tissue) using a 200× magnification. At two measurement points from each visible muscle fiber, the width of the muscle fiber cylinder was determined in μm, and then the average of all measurements was calculated. The measurements were performed perpendicular to the axis of the muscle fiber (Figure 2). 

Visible muscle tissue was quantified by calculating the muscle tissue fraction, defined as the ratio of muscle tissue to total tissue in a section. A HE muscle section was imaged in its entirety at 400× magnification using Olympus cell^D software, followed by digital processing in Photoshop CS4 version 11.0.2 using the “magic wind” function. This allowed precise identification and pixel count of muscle cells. The muscle tissue fraction was then calculated using the total tissue pixel count.

### 2.10. Statistical Analysis

All data are presented in box plots with the median value, 1st and 3rd quartile, and whiskers with the minimum and maximum values. If the values were normally distributed (evaluated using the Shapiro–Wilk test), the one-way analysis of variance was used; otherwise, an ANOVA on ranks was performed. Statistical testing was performed using SigmaPlot 13.0 software (Jandel Corp., San Rafael, CA, USA). Differences with *p* < 0.05 were considered significant.

## 3. Results

### 3.1. General Observations

All animals successfully completed the experiment. Following the induced muscle trauma, the animals used their lower left limbs less for a few days. Surgical wounds healed normally, with no complications or signs of local infection. The right hind leg, which was not exposed to muscle injury, showed no functional limitations in any of the experimental groups.

### 3.2. Muscle Force

**Figure 3:**
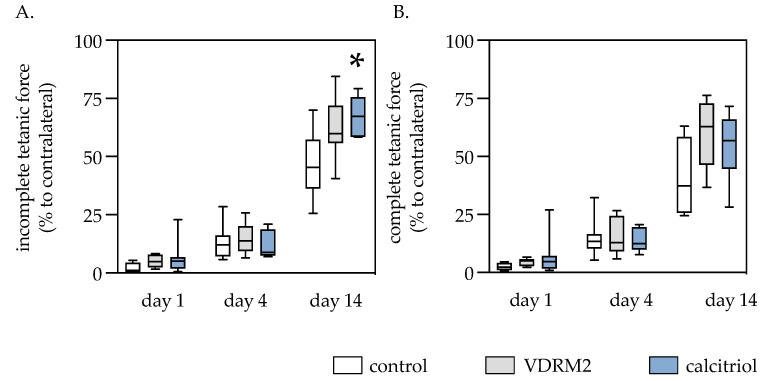
Quantitative analysis of the incomplete tetanic force (**A**) and the complete tetanic force (**B**) of the injured left soleus muscle, expressed relative to the force of the contralateral non-injured soleus muscle (in %). The animals underwent after-muscle injury treatment with VDRM2, calcitriol, or equivalent volumes of 10% ethanol (control). The data are presented as box plots (median, 1st, and 3rd quartile) with whiskers (minimum and maximum values). Statistical analysis: one-way ANOVA; * *p* < 0.05 vs. control.

Upon injury, there was a substantial decline in muscle force followed by a gradual improvement in the incomplete tetanic muscle contraction force in all three groups. No significant difference was noted in the incomplete tetanic muscle force of the left soleus muscle among the groups on the first and fourth day after trauma. However, fourteen days post-trauma, the group treated with calcitriol showed a significant increase in incomplete tetanic contraction compared to the control group. In the calcitriol group, there was a 36% decline in contractile force after 14 days. At the same time point, the VDRM2 and control groups declined muscle strength by 37% and 52%, respectively. Muscle force measurement of the complete tetanic force showed a similar kinetic to the incomplete tetanic contraction. Notable but not significant differences between the calcitriol, VDRM2, and control groups emerged on the 14th day, with the first two groups showing higher complete tetanic contraction values (Figure 3).

### 3.3. Proliferation

**Figure 4:**
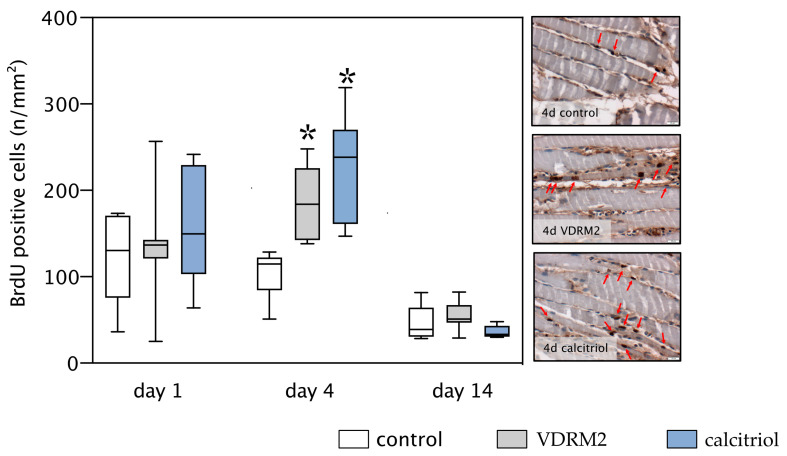
Quantitative analysis of the BrdU-positive cells (**left panel**) and exemplary images of BrdU staining at day 4 (**right panel**) of the injured left soleus muscle. Positive cell nuclei are stained brown and are pointed out by red arrows. Deep blue cell nuclei did not incorporate BrdU; muscle cells are illustrated in a light blue-brownish color. The animals underwent after-muscle injury treatment with VDRM2, calcitriol, or equivalent volumes of 10% ethanol (control). The data are presented as box plots (median, 1st, and 3rd quartile) with whiskers (minimum and maximum values). Statistical analysis: one-way ANOVA; * *p* < 0.05 vs. control.

Proliferative activity within the control group was notably elevated at days 1 and 4 post-injury and almost diminished by day 14. In contrast, the administration of either VDRM2 or calcitriol led to a significant increase in muscle cell proliferation on day 4 compared to the control group. However, there were no detectable differences between all experimental groups on day 1 and day 14 post-injury (Figure 4).

### 3.4. Apoptosis

**Figure 5:**
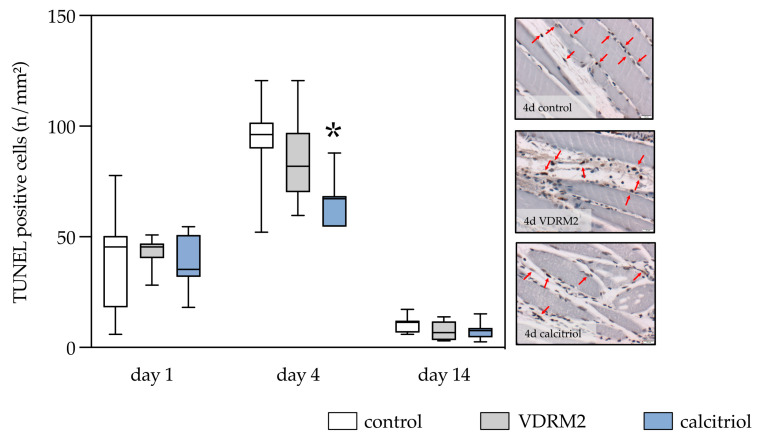
Quantitative analysis of the TUNEL-positive cells (**left panel**) and exemplary images of TUNEL staining at day 4 (**right panel**) of the injured left soleus muscle. Positive cell nuclei are stained deep red and are pointed out by red arrows. Blue cell nuclei did not incorporate BrdU. The animals underwent after-muscle injury treatment with VDRM2, calcitriol, or equivalent volumes of 10% ethanol (control). The data are presented as box plots (median, 1st, and 3rd quartile) with whiskers (minimum and maximum values). Statistical analysis: one-way ANOVA; * *p* < 0.05 vs. control.

The control group displayed an apoptotic trajectory characterized by high values on day 1, peaking on day 4, and markedly decreasing by day 14. Almost all TUNEL-positive cells were located interstitially. A similar pattern was observed in both the VDRM2 and calcitriol groups. Notably, the calcitriol group showed a significant reduction in apoptosis on day 4 compared to the control group, with the VDRM2 group displaying intermediate values. Days 1 and 14 revealed minimal intergroup differences in apoptotic cell counts within the traumatized left muscle (Figure 5).

### 3.5. CAE Positive Cells

**Figure 6:**
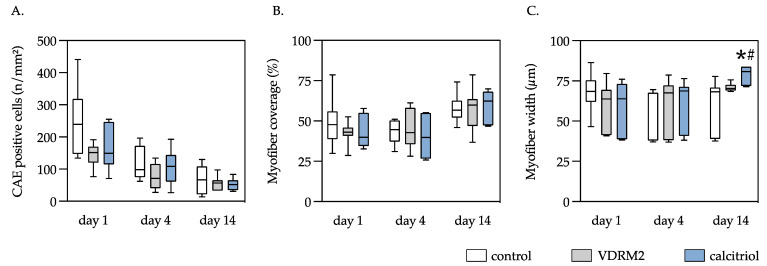
Quantitative analysis of the CAE-positive cells (**A**), myofiber coverage (**B**), and myofiber width (**C**) of the injured left soleus muscle. The animals underwent after-muscle injury treatment with VDRM2, calcitriol, or equivalent volumes of 10% ethanol (control). The data are presented as box plots (median, 1st, and 3rd quartile) with whiskers (minimum and maximum values). Statistical analysis: one-way ANOVA; * *p* < 0.05 vs. control, ^#^ *p* < 0.05 vs. VDRM2.

In the control group, exceedingly high tissue infiltrating mast cells, myeloid cells, and neutrophils quantified using CAE-staining were observed on day 1, with considerable counts on day 4 and markedly diminished counts on day 14. This temporal pattern was mirrored in both the VDRM2 and calcitriol groups. Statistical analysis across time points revealed no significant disparities among the VDRM2, control, and calcitriol groups (Figure 6A).

### 3.6. Myofiber Width and Muscle Tissue Fraction

On day 14, the myofiber width in the calcitriol group showed a significant increase compared to the control and VDRM2 groups. Variations in myofiber width on days 1 and 4 among the groups were not significant. The muscle tissue fraction, which offers indirect insights into tissue damage extent, did not show a difference between the control and the VDRM2 and calcitriol-treated groups. However, an increase in the intact muscle area was noted over time in all groups. On day 14, approximately 56.2% of the muscle tissue had regenerated (Figure 6B,C).

## 4. Discussion

### 4.1. Methodological Considerations

While many researchers analyze muscle tissue using cross-sections for histology, it may not be suitable for our study. Our soleus muscle model exhibits alternating patterns of heavy and lightly injured muscle tissue [17]. This inconsistency necessitates longitudinal sections, allowing objective muscle tissue analysis. Relying on cross-sections poses a risk, as they might predominantly capture regions of either severe or minor injury, potentially affecting the accurate measurement of proliferation and apoptosis based on where the tissue is sectioned [18]. Additionally, the muscle fiber width measurements from longitudinal sections should not be equated with the myofiber diameter derived from muscle cross sections. This inconsistency is due to the lack of studies that have concurrently investigated muscle width in longitudinal sections and the cross-sectional area of the same muscle.

### 4.2. Calcitriol, VDRM2, Muscle Degradation and Regeneration

Calcitriol status has a crucial role in muscle damage and regeneration. The mechanism of action was studied in the past [6]. Calcitriol deficiency can lead to various cascades and pathologic conditions, including mitochondrial dysfunction, diminished ATP production, increased reactive oxygen species production, oxidative damage, muscle wasting, and impaired muscle function. These conditions could potentially exacerbate symptoms associated with muscle damage [6]. During muscle regeneration, vitamin D stimulates an increase in the vitamin D receptor in the satellite cells. These changes in the VDR are accompanied by a stimulation of satellite cell differentiation [6]. Moreover, VDR signaling promotes mitochondrial biogenesis and fusion signaling, inhibits ROS production, and could attenuate the need for antioxidants, potentially contributing to an enhanced regenerative phenotype [6].

Our results show that both calcitriol and VDRM2 significantly increased the number of proliferating cells four days post-trauma. The effect of calcitriol on cell proliferation, however, has been found to vary across different types of tissues. For instance, its application appears to hinder the proliferation of skin’s keratinocytes [19] while enhancing their differentiation, an effect already employed clinically, for example, in psoriasis and atopic dermatitis [20]. For the skeletal muscle, the evidence points to a significant influence of calcitriol on satellite cell functionality in preserving skeletal muscle homeostasis [21]. In contrast, certain benign and malignant tumors show in vitro and in vivo reduced proliferation upon calcitriol administration [22]. The differential effects of calcitriol may be attributable to structural variations in the calcitriol receptor (VDR) across various tissues, as reported by Campbell et al. [23]. Such variations could lead to different signaling cascades, accounting for the conflicting evidence on calcitriol’s proliferative effects, including in skeletal muscle tissues [6]. Recent research suggests that calcitriol influences muscle repair by modulating satellite cell proliferation and differentiation, as well as mitochondrial density and function [6]. In earlier research, VDRM2 exhibited antiproliferative effects on skin and prostate epithelia [14,24]. Other calcitriol analogs also displayed similar antiproliferative effects, such as in melanoma cells [25]. This study, however, is the first to document a pro-proliferative effect of VDRM2 on muscle tissue, thereby suggesting its tissue-specific action, much like calcitriol itself. Our previous research [5], which utilized the same experimental setup and a comparable dose of Vitamin D, showed that applying Vitamin D during muscle injury does not influence the proliferation of satellite cells, denoted using PAX-7 positive cells. Due to the lack of proliferative activity of satellite cells after vitamin D application, it is reasonable to assume that VDRM2 also has no proliferative activity on the satellite cells. Our present also support this hypothesis. VDRM2 had no effect on muscle strength, muscle tissue area, or muscle fiber width. If there was an increase in satellite cell numbers, these metrics would also increase. Both our past results and present data reinforce the understanding that the observed functional restoration is primarily driven by a rise in cellular turnover, marked by an increase in proliferation and a decrease in apoptosis. Further investigation is needed to ascertain whether these effects extend to calcitriol analogues, particularly VDRM2, emphasizing the complex interplay between calcitriol, its receptors, and cell proliferation in various tissue types.

The mechanisms through which calcitriol influences apoptosis seem to vary widely across different types of cells. For instance, calcitriol has been proposed to modulate apoptosis via calcium signaling in both mammary adipocytes and mammary tumor cells, as suggested by a study [26]. Moreover, calcitriol has been involved in the DNA repair process. It has been suggested to regulate both apoptosis and autophagy in cancer cells and also seems to influence various signaling pathways associated with tumorigenesis [27]. This complex role of calcitriol in cancerous cells underscores the importance of context when considering its effects on apoptosis. Diversely, in the context of human nucleus pulposus cells, calcitriol acts to inhibit apoptosis triggered by TNF-α, accomplishing this using the regulation of the NF-kB signaling pathway. Furthermore, in the case of corneal epithelial cells under hyperosmotic stress, calcitriol has been demonstrated to inhibit apoptosis via the activation of autophagy, both in vitro and in vivo [28]. Finally, there’s evidence supporting the beneficial role of calcitriol in cases of skeletal muscle injury. Specifically, previous research has suggested that calcitriol administration can reduce apoptotic cell death in injured skeletal muscle in rats [5]. In summary, calcitriol’s influence on apoptosis is complex, and for most cell types and pathologic conditions, calcitriol has an antiapoptotic action.

It is widely accepted that calcitriol can help reduce inflammation in skeletal muscle, and meanwhile, calcitriol is identified as an efficacious anti-inflammatory agent in numerous studies [29]. Choi et al. highlight the modulatory effect of calcitriol supplementation on inflammatory responses, particularly in the context of muscle damage provoked by high-intensity exercise, as studied in rat models [30]. In our current study, we observed a measurable but not statistically significant reduction in CAE-positive cells on the first day following muscle injury, which was induced using calcitriol and VDRM2. A possible interpretation of this finding points to the dose-dependent nature of calcitriol’s response to inflammation since it is possible that larger doses of calcitriol may amplify its anti-inflammatory action, thereby inducing a more pronounced effect [31].

### 4.3. Dose of Calcitriol and VDRM2

The use of VDRM2, particularly in the context of skeletal muscle, is still a new avenue due to its recent introduction. Sato et al. [15] have shown in their work that rats could tolerate VDRM2 up to a dosage of 4.6 μg/kg body weight before any hypercalcemic side effects were observed. Interestingly, a positive effect on bone strength was evident at much lower dosages, ranging from 0.1 to 3 μg/kg body weight [15]. In the current experimental setup, we aimed to explore the potential influence of VDRM2 on muscle repair. To establish this initial investigation, we chose to administer a dosage of 1 μg/kg body weight. In our previous research, we found that subcutaneous doses of 0.83 mg/kg and 8.3 mg/kg body weight did not trigger hypercalcemia, hypercalciuria, or reduction in parathyroid hormone levels throughout their examination period [5]. For this study and to ensure a fair comparison between the effects of VDRM2 and calcitriol, a dose of 1 μg/kg body weight was selected for intraperitoneal administration. This consistent dosing strategy across both compounds aids in the reliability and comparability of the experimental findings.

### 4.4. Muscle Strength

This study explored the impact of trauma on the soleus muscle, identifying a reduction in relative muscle strength as a primary outcome. However, it was found that muscle strength could be significantly enhanced with the application of calcitriol and also moderately improved using the administration of VDRM2. The positive effect of calcitriol on muscle strength is well documented and corroborated by multiple studies [32]. Calcitriol has been shown in clinical studies to enhance muscle strength and function and even reduce the risk of falls post-application [33,34]. Its ability to influence muscle regeneration positively has been further evidenced in both in vitro and in vivo experiments [5]. The inclusion of VDRM2 in this context is relatively novel. From a molecular standpoint, it is plausible to infer that both calcitriol and VDRM2 exert their effects via the activation of similar genes. This hypothesis is supported by other studies using RNA sequencing, revealing that calcitriol and VDRM2 modulate about 80% of the same genes, either upregulating or downregulating them [14]. Notably, VDRM2 seems to have less transcriptional strength and induce fewer signaling cascades than calcitriol. Yet, its advantage lies in its extended availability. Unlike calcitriol, VDRM2 is not degraded by the cytochrome P450 24A1 (CYP24A1), thereby ensuring its sustained presence [9,14]. Nevertheless, while these findings suggest a beneficial role for VDRM2 in muscle trauma recovery, it remains necessary to conduct further studies to elucidate its exact mechanism of action.

The difference between complete (fused) and incomplete (unfused) tetanic contraction lies in the level of muscle activation and the pattern of muscle fiber recruitment. Complete tetanic contraction occurs when the muscle fibers are fully activated, and there is no relaxation between consecutive stimuli, while unfused tetanic contraction occurs when there is some degree of relaxation between stimuli [35,36]. Fused contractions are characterized by sustained force output, while unfused contractions are characterized by twitch-like force output with fluctuations in force. Complete tetanic contractions are more economical in terms of cross-bridge interaction and heat production compared to unfused contractions.

One potential reason for the notable contrast in the incomplete tetanic force on day 14, while at the same time, no significant difference is observed in the tetanic force, may be attributed to the interplay between muscle fiber activation and mechanomyogram (MMG) signals. Bichler [37] suggests that the number of active muscle fibers, their topography, and their localization in relation to the muscle surface influence MMG phenomena. This suggests that incomplete tetanic contractions, which involve the activation of different groups of motor units in rotation, may result in a more diverse and widespread activation of muscle fibers, leading to significant MMG signals.

## 5. Conclusions

The results of this study demonstrate that the systemic application of calcitriol upon muscle injury leads to increased cellular turnover with increased proliferation and reduced apoptosis, as well as an increase in muscle force and myofiber width. Additionally, the use of VDRM2 shows a slight enhancement in muscle proliferation and a minor but not statistically significant improvement in muscle force. While these results cannot be directly extrapolated to human application without further investigation, calcitriol exhibits more promise compared to VDRM2. The demonstrated effects indicate that calcitriol may facilitate improved force recovery following muscle injury, as evidenced by the data on increased incomplete tetanic force and myofiber width by day 14 post-injury.

## Data Availability

The data generated during the current study are available from the corresponding author upon reasonable request.
